# Retinol and Oligopeptide-Loaded Lipid Nanocarriers as Effective Raw Material in Anti-Acne and Anti-Aging Therapies

**DOI:** 10.3390/life14101212

**Published:** 2024-09-24

**Authors:** Małgorzata Pawłowska, Marta Marzec, Waldemar Jankowiak, Izabela Nowak

**Affiliations:** 1 Department of Applied Chemistry, Faculty of Chemistry, Adam Mickiewicz University, Poznań, Uniwersytetu Poznańskiego 8, 61-614 Poznań, Poland; malgorzata.pawlowska@amu.edu.pl; 2 Dottore Polska Sp. z o.o., Margonińska 22, 60-425 Poznań, Poland

**Keywords:** solid lipid nanoparticles, retinol, oligopeptide, anti-aging therapy, anti-acne action

## Abstract

The use of lipid nanocarriers as components of cosmetic formulations may provide an opportunity to fully exploit the beneficial properties of pentapeptide-18 and retinol while reducing the undesirable effects that occur during retinoid therapy. This study aimed to evaluate the effectiveness of semi-solid formulations enriched with retinol and oligopeptide-loaded lipid nanocarriers. Solid lipid nanoparticles were produced using a high-shear homogenization method. The work included physicochemical characterization of the cosmetic products, and evaluation of their stability as well as their efficacy. The resulting semi-solid preparations were determined to be stable regardless of their storage temperature. No effect of the presence of lipid nanoparticles on the shelf-life stability of the cosmetic products was observed. A temperature of 25 °C was considered the recommended storage temperature for the tested semi-solid formulations. Beneficial effects of the cosmetic products were proven (in vivo study on volunteers), i.e., a significant reduction in the level of sebum secretion (anti-acne therapy) and a decrease in the number of facial wrinkles (anti-aging therapy). In addition, the protective properties of the lipid nanoparticles themselves against the skin were confirmed, reducing the irritating effect of retinol that is usually the case with classic retinoid therapies.

## 1. Introduction

Harnessing the therapeutic potential of retinol and peptides in line with the current trend of “skin minimalism” is possible through the use of solid lipid nanoparticles (SLNs), colloidal carriers of active substances for topical application [1]. Lipid nanoparticles provide a delivery system for active compounds, increasing their bioavailability while controlling permeation and distribution in target tissues [2]. The biocompatibility and lack of toxicity of the aforementioned lipid carriers are primarily related to the structure of the lipid matrix, which undergoes enzymatic degradation to compounds naturally found in the human body [2].

Peptides used in cosmetics have a low molecular weight and are hydrophilic in nature [3]. Based on their structure and chain length, these compounds can be divided into oligopeptides, polypeptides, and proteins [4,5]. The study of Olejnik et al. [5] showed that the group of peptides used in cosmetics is also divided in terms of action and function concerning the skin–signal peptides, so-called neuropeptides, and carrier peptides. The oligopeptide (pentapeptide-18) used in the study belongs to the group of neurotransmitter inhibitor peptides, which in its action resembles the natural enkephalin pathway by affecting the reduction in acetylcholine release at the synapse in the nervous system [6,7]. Clinical studies indicate its broad anti-aging effect on the skin, including by reducing the depth of facial wrinkles [6].

Retinol, a compound from the retinoid group, shows analogous properties and structure characteristics of vitamin A [8]. Retinol is the primary biologically inactive form and must be converted to retinal and then to retinoic acid, which is the only biologically active form [9]. This process occurs in the cell and is associated with RBPs (retinoid binding proteins) [10,11]. The positive effects of retinol can be observed in all layers of the skin (collagen synthesis and regulation of sebaceous glands) [12].

The incorporation of retinol into a matrix of lipid nanoparticles may provide a solution to the problematic limitations of retinoids when they are used in cosmetic therapies. Wissing and Lasoń et al. [13,14] pointed out the contribution of solid lipid nanoparticles to the controlled release of active substances and to protecting them from chemical degradation; this includes retinol and peptides. Also emphasized are the occlusive properties of lipid nanoparticles, which increase skin hydration [15]. Ferreira et al. [16] further pointed out that retinoids delivered topically in the form of lipid nanoparticles are more stable and better tolerated by the skin. Anil and Kannan [17], on the other hand, stressed that the incorporation of peptides inside lipid nanoparticles provides better bioavailability, while Almeida et al. [18] pointed out the increased stability, prolonged release of peptides, and prevention of their degradation.

Nowadays, the cosmetic market offers many active substances dedicated to the care of aging skin, where ensuring an adequate level of hydration and effective antioxidant activity play an important role [19]. Among the most commonly used active compounds, in addition to the aforementioned retinol and peptides, are vitamin C [19,20] and glycolic acid [21]. These substances have an anti-aging effect through their antioxidant and exfoliating properties. On the other hand, in the case of acne-prone skin and skin with imperfections, chemical peels with the use of, among other components, mandelic and pyruvic acids, are considered to be the most effective. They have a beneficial effect on the regulation of the sebaceous glands, while also having an antibacterial effect, which are desired for the care of skin with acne problems [22,23].

The purpose of the study presented in this paper was to introduce the designed dispersion of lipid nanoparticles, i.e., solid lipid nanoparticles loaded with retinol and pentapeptide-18 (oligopeptide, Tyr-D-Ala-Gly-Phe-Leu), into semi-solid cosmetic preparations in the form of night face creams. The work included physicochemical characterization of the cosmetic products, and evaluation of their stability as well as their efficacy (in vivo studies). The novelty of this research lies in the combination of retinol and oligopeptide in a lipid carrier, which is a new solution unprecedented in the cosmetic market. An attempt was also made to verify the effectiveness of the anti-aging oligopeptide and the reduction in the irritant potential of retinol introduced into lipid carriers and used in anti-aging and anti-acne therapies.

## 2. Materials and Methods

### 2.1. Production and Characterization of Lipid Nanoparticles

**Figure 1:**
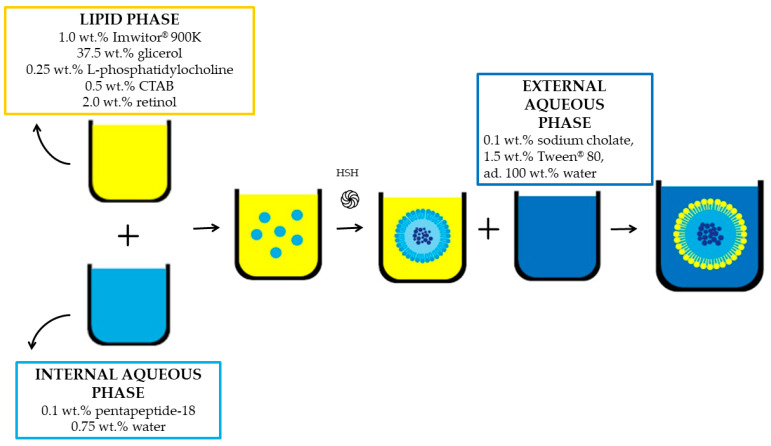
**A** step-by-step scheme for production of lipid nanoparticles.

Lipid nanoparticles were produced by applying a high shear homogenization (HSH) method based on multiple emulsion (W/O/W) (Figure 1). The lipid phase (1.75 wt.%)—L-phosphatidylcholine (Sigma-Aldrich, Steinheim, Germany), Imwitor^®^ 900 K (glyceryl monostearate; IOI Oleo GmbH, Hamburg, Germany), and hexadecyltrimethylammonium (CTAB; Chemat, Gdańsk, Poland)—was prepared first, and heated to the melting point of the lipid. The next step was to add 2.0 wt.% of retinol (DSM, Kaiseraugst, Switzerland) and then glycerol (Chempur, Piekary Śląskie, Poland). At the same time, an aqueous solution of Tween^®^ 80 at a concentration of 3% *w*/*w* (polysorbate 80; Pol-Aura, Zawroty, Poland) was prepared with the addition of 0.1 wt.% sodium cholate (Acros Organics, Antwerp, Belgium). Leuphasyl^®^ peptide (pentapeptide-18/Tyr-D-Ala-Gly-Phe-Leu; Lipotec, Barcelona, Spain), which was the internal aqueous phase, was added to the lipid phase (high shear homogenization, 8000 rpm/15 min–Ultra-Turrax^®^ DI 25 Basic, IKA-Werke GmbH, Staufen im Breisgau, Germany). The final step was to add the resulting pre-emulsion to the previously prepared aqueous surfactant solution under continuous stirring.

A set of physicochemical parameters of lipid nanoparticle dispersions—mean particle size (Z-Ave), polydispersity index (PDI), and zeta potential (ZP)—was determined with a Zetasizer Nano ZS (Malvern Instruments, Malvern, UK). These parameters were determined based on the scattering of the laser light beam generated by the device on the particles of the dispersion of lipid nanoparticles, with respect to the following phenomena: (i) dynamic light scattering (DLS)–Z-Ave&PDI: the method is based on measuring changes in the intensity of light scattered under the influence of the movement of dispersed particles; and (ii) electrophoretic light scattering (ELS)–ZP: a technique based on measuring the velocity of dispersion particles moving in an electric field generated during the electrophoresis process [24]. Before the measurements, aqueous solutions of the tested lipid nanoparticle dispersions were prepared, namely, 60 µL of the dispersion was dissolved in 15 mL of distilled water. The measurement procedure was repeated three times for each test sample, and the arithmetic mean and standard deviation were calculated from the obtained results.

### 2.2. Preparation of Semi-Solid Cosmetic Formulations

The study was performed on four night-type face creams produced by Dottore Polska Sp. z o.o., Poznan, Poland, labeled as follows:

- AAN (anti-aging NANO)—anti-aging night face cream containing lipid nanoparticles loaded with retinol and pentapeptide-18 (10.0 wt.%);
- AAB (anti-aging BASE)—anti-aging night face cream without lipid nanoparticles;
- ACN (anti-acne NANO)—anti-acne night face cream containing lipid nanoparticles loaded with retinol and pentapeptide-18 (5.0 wt.%);
- ACB (anti-acne BASE)—anti-acne night face cream without lipid nanoparticles.

The technology of the production of the above cosmetic bases (AAB and ACB) remains the know-how of the manufacturer (Dottore Polska Sp. z o.o.). Briefly, the emulsions are of *o*/*w* type with 21.2 wt.% of lipid phase and were formed by using a homogenization process. The introduction of lipid nanoparticles into base products (AAB and ACB) consisted of combining the cosmetic base with an appropriate amount of lipid nanoparticle dispersion under continuous stirring. The process continued until the appropriate consistency of cosmetic formulations enriched with lipid carriers (AAN and ACN) was achieved. The INCI compositions of night face creams investigated are shown below. The ingredients were grouped according to their content in the cosmetic formulation and assigned to one of the following ranges: (A) 50–100 wt.%; (B) 25–50 wt.%; (C) 10–25 wt.%; (D) 5–10 wt.%; (E) 1–5 wt.%; (F) 0.1–1 wt.%; (G) <0.1 wt.%; (H) traces.

AAN (anti-aging NANO)

Ingredients (INCI): Aqua (A), Tripelargonin (D), Glycerin (D), Isopropyl Palmitate (D), Polyglyceryl-3 Dicitrate/Stearate (E), Persea Gratissima Oil (E), Glyceryl Stearate (E), Retinol (F), Pentapeptide-18 (G), Tocopheryl Acetate (F), Xanthan Gum (F), Benzyl Alcohol (F), Benzoic Acid (F), Polysorbate 80 (F), Dehydroacetic Acid (G), Tocopherol (G), Phosphatidylcholine (G), Cetrimonium Bromide (G), Sodium Cholate (G), Sodium Hydroxide (H), Tetrasodium Glutamate Diacetate (G), Parfum (F)

AAB (anti-aging BASE)

Ingredients (INCI): Aqua (A), Tripelargonin (D), Glycerin (D), Isopropyl Palmitate (D), Polyglyceryl-3 Dicitrate/Stearate (E), Persea Gratissima Oil (E), Glyceryl Stearate (E), Tocopheryl Acetate (F), Xanthan Gum (F), Benzyl Alcohol (F), Benzoic Acid (F), Dehydroacetic Acid (G), Tocopherol (G), Sodium Hydroxide (H), Tetrasodium Glutamate Diacetate (G), Parfum (F)

ACN (anti-acne NANO)

Ingredients (INCI): Aqua (A), Citrullus Lanatus Seed Oil (D), Propanediol (E), Citrus Limon Fruit Extract (E), Isopropyl Palmitate (E), Glyceryl Stearate Citrate (E), Cetearyl Alcohol (E), Pentylene Glycol (E), Glyceryl Stearate (E), Squalane (E), Cannabis Sativa Seed Oil (E), Glycerin (E), Sodium PCA (E), Succinoglycan (F), Pentapeptide-18 (G), Retinol (F), Phosphatidylcholine (G), Cetrimonium Bromide (G), Sodium Cholate (G), Polysorbate 80 (F), Cannabidiol (G), Bioflavonoids (G), Raspberry Ketone (F), Tocopherol (G), Helianthus Annuus Seed Oil (F), Potassium Sorbate (G), Tetrasodium Glutamate Diacetate (G), Sodium Hydroxide (H), Trisodium NTA (H), Parfum (F)

ACB (anti-acne BASE)

Ingredients (INCI): Aqua (A), Citrullus Lanatus Seed Oil (D), Propanediol (E), Citrus Limon Fruit Extract (E), Isopropyl Palmitate (E), Glyceryl Stearate Citrate (E), Cetearyl Alcohol (E), Pentylene Glycol (E), Glyceryl Stearate (E), Squalane (E), Cannabis Sativa Seed Oil (E), Glycerin (E), Sodium PCA (E), Succinoglycan (F), Cannabidiol (G), Bioflavonoids (G), Raspberry Ketone (F), Tocopherol (G), Helianthus Annuus Seed Oil (F), Potassium Sorbate (G), Tetrasodium Glutamate Diacetate (G), Sodium Hydroxide (H), Trisodium NTA (H), Parfum (F)

The effectiveness of the production method for the semi-solid cosmetic formulations was verified on a production scale. The stability of both the key raw material (lipid nanoparticle dispersion) and the finished cosmetic products was confirmed. The production method was developed in accordance with all regulatory standards for the concentrations of raw materials used. In addition, the high-speed homogenizer used within the framework of the study is a device commonly found in cosmetic manufacturing companies, which does not generate additional difficulties at the various stages of production of the developed cosmetic products. Furthermore, the method for obtaining lipid nanoparticles was prepared in such a way as not to cause limitations in the production of SLNs with retinol and oligopeptide on a production scale. The effective transfer of production from laboratory scale to industrial scale was a strong point of the implementation PhD under which this research was conducted, and confirmed the translational significance of the study.

### 2.3. Physicochemical Characterization of Cosmetic Products

#### 2.3.1. pH

The pH (EcoSense^®^ pH 10 pH/Temperature Meter, Pen Style; VWR International, Radnor, PA, USA) of the cosmetic products was tested on the day of their production (week 0, denoted as W0) and after 2 months (week 8, denoted as W8) of storage. The pH test was conducted for samples stored at three temperatures (4, 25, and 45 °C). The measurement procedure was repeated three times for each test sample, and the arithmetic mean and standard deviation were calculated from the obtained results.

#### 2.3.2. Stability Test

The stability of the cosmetic products was evaluated by the method of multiple light scattering using a Turbiscan Lab Expert analyzer (Formulation, L’Union, France). Measurements were made on day 0 and then repeated on the 7th, 30th, and 60th days for the samples stored at three temperatures (4, 25, and 45 °C). The measurement procedure was repeated three times for each test sample. The results were obtained as the curves illustrating changes in the light backscattering (BS) and the Turbiscan Stability Index (TSI) calculated by the device software.

### 2.4. The Application Tests

The application tests lasted 8 weeks, and measurements of biophysical parameters of the skin were taken on the day of the start of the tests (W0) and at weeks 4 and 8 (W4 and W8). All volunteers were informed about the nature of the study, signed an informed consent form to participate in the tests, and received written informational materials about the use of the cosmetic products being tested. The participants completed a questionnaire on their health status, possible skin problems, and skin care habits. A detailed cosmetic interview was also conducted. Each measurement was taken after a 15 min acclimatization in the examination room; this was done to equalize skin temperature and regulate sweat secretion in the volunteers, which in turn translated into the accuracy and reliability of the measurement. In the 8th week of the study, all volunteers filled out a subjective questionnaire about the application sensations and effectiveness of the tested products. Any adverse effects were to be reported to the investigator at any stage of the application tests. The in vivo study was approved by the Bioethical Commission of Poznan University of Medical Sciences, Poland, on 12 October 2023 (768/23). The Wilcoxon test for pairs of observation results was used for statistical analysis. The level of significance was *p* < 0.05.

#### 2.4.1. Testing Panel

The testing panel consisted of two groups of 20 female volunteers. The selection criteria for the first group (anti-aging, AA), in the 30–65 age range, were signs of skin aging, i.e., wrinkles and loss of skin elasticity and firmness. For the second group (anti-acne, AC), in the 18–65 age range, the criteria were imperfections and skin inflammation, with an emphasis on the acne substrate of the lesions. Each volunteer received two cosmetic products (night face creams) for testing: group AA—cream AAN (anti-aging NANO) and cream AAB (anti-aging BASE); group AC—cream ACN (anti-acne NANO) and cream ACB (anti-acne BASE). The volunteers undertook to apply the tested cosmetic products to cleanse facial skin for the entire duration of the study, once a day, exclusively at night. All participants were informed that they were prohibited from using products with similar or analogous ingredients or actions. Information was also given on the need to avoid sun exposure, including tanning beds, due to the nature of the active substances contained in the tested cosmetic product. The main contraindications to participation in the study, which were pregnancy and lactation, were also mentioned. 

#### 2.4.2. Skin Testing Equipment

Evaluation of selected biophysical parameters of the skin was carried out using a set of equipment from Courage + Khazaka electronic GmbH (Cologne, Germany). The equipment, using the technique of non-invasive evaluation of skin parameters, made it possible to observe changes appearing under the influence of active substances (retinol and oligopeptide contained in the lipid nanoparticles) present in the tested cosmetic products. The devices used in the study were:

- Tewameter^®^ TM 300 (level of transepidermal water loss, TEWL)

The Tewameter^®^ TM 300 allows assessment of the functioning of the epidermal barrier. Transepidermal water escape is a natural process occurring in the epidermis, but changes in the biophysical state of the skin and the application of cosmetic products can modify the parameters of this process. The probe works by measuring the density of water evaporation from the skin, based on the law of diffusion [25]. The lower the level of water evaporation, and thus the lower the measurement result, the better the tightness of the epidermal barrier. The measurement was performed by applying the probe vertically to the skin surface of the forehead and cheek, on the left and right sides of the face. Twenty measurements were taken on each area of the volunteers’ facial skin. The arithmetic mean was calculated from the values obtained. Measurements were taken on the day of the start of the study (W0) and after 4 and 8 weeks of application of the tested cosmetic products (W4 and W8).

- Corneometer^®^ CM 825 (skin hydration level)

The Corneometer^®^ CM 825 is a probe that determines the moisture level of the skin. The measurement is based on the physical electrical conductivity of the tissues and the electrical resistance they generate. Due to the large amount of water in the epidermal cells, and thus due to proper skin hydration, the electrical resistance decreases, while the electrical conductivity increases. Thus, the higher the measurement, the better the hydration of the skin [26]. The results were collected in volunteers from the left and right facial areas of the forehead and cheek by vertically applying the probe to the skin surface. The test was conducted at W0, W4, and W8, where three measurements were taken each time, from which the arithmetic mean was calculated.

- Cutometer^®^ MPA 580 (skin elasticity)

The Cutometer^®^ MPA 580 is a device used to measure the viscoelastic properties of the skin. The probe measures the elasticity and firmness of the upper layer of the skin using negative pressure. The suction method causes mechanical deformation of the skin. Its resistance to vacuum determines firmness, while its ability to return to its original shape indicates elasticity. This method makes it possible to objectively assess the aging process of the skin and to generally characterize the elasticity and mechanical properties of the epidermal surface [27]. The general elasticity factor (R2) was determined before cosmetic product application (W0) and after 8 weeks (W8), using a Cutometer^®^ that was applied to the facial surface without additional pressure. This allowed the skin to be sucked into the opening, where a vacuum was generated. During each measurement session, three measurements were taken on each test area of the skin. The arithmetic mean was calculated from the results obtained.

- Sebumeter^®^ SM 815 (amount of sebum secreted by the skin’s sebaceous glands)

Sebum secretion is a natural physiological process of the skin. The Sebumeter^®^ SM 815 measures the amount of sebum secreted on the epidermal surface. Sebum from the surface of the skin is collected using a special tape that becomes transparent under the influence of sebum. The tape is placed in a designated hole in the base of the MPA device, where a beam of light, transmitted through the section of tape being tested, allows the level of its saturation to be read [28]. The study was conducted on a group of volunteers using anti-acne products (ACN and ACB). The tape was applied at four locations of skin: on the left and right cheek, and on the left and right sides of the forehead at a height of about 1 cm above the upper eyebrow arch. Sebumeter^®^ measurements were taken on the day the study began (W0) and at W4 and W8.

- Visioline^®^ VL 650 (skin macrorelief parameters: total wrinkle area [mm^2^]; percentage of wrinkle area [%]; mean length [mm] and maximum depth of wrinkles [µm])

Skin macrorelief parameters were determined on the basis of images of silicone replicas taken on the skin areas under study. The Visioline^®^ VL 650 works on the principle of profilometry: a light beam falls on the replica at an angle of 35° and makes visible the elevations of the replica, which represent the wrinkles of the skin. The measurable shadows are digitized by the camera and processed using software [29]. Silicone skin replicas were made on the skin of volunteers (forehead and cheeks on both sides of the face) using anti-aging creams (AAN and AAB) on the day the tests began (W0) and after 8 weeks (W).

## 3. Results and Discussion

### 3.1. Lipid Nanoparticles Loaded with Retinol and Pentapeptide-18–Physicochemical Characterization

The desire to introduce lipid nanoparticles as a cosmetic raw material in the production of cosmetic preparations on an industrial scale is an important aspect when planning and optimizing the composition of designed lipid nanocarriers. The stability of nanoparticle dispersion significantly affects the effectiveness of the incorporated active substances [18]. Moreover, the presence of the incorporated active compounds positively affects the stability of the dispersion itself. The selection of individual dispersion components depends on the nature of the incorporated active substance. On the other hand, the wrong concentration of lipids (both solid and liquid) and surfactants can affect the overall internal structure of the lipid nanoparticles [15]. Proper selection of physicochemical parameters for lipid nanoparticle dispersion can also help predict SLNs’ behavior in in vivo studies [15]. When lipid nanocarriers are introduced into cosmetic formulations, it is necessary to maintain dispersion stability for a minimum of four weeks [30]. For the prepared dispersion of solid lipid nanoparticles containing oligopeptide and retinol, the following values of physicochemical parameters were obtained: Z-Ave = 156.5 ± 0.8 nm; PDI = 0.284 ± 0.002; and ZP = 45.6 ± 0.2 mV. The long-term stability of the lipid nanoparticles was tested by storing the dispersion samples at three temperatures (4 °C, 25 °C, and 45 °C) for one month. After four weeks, the following results were obtained: at 4 °C: Z-Ave = 248.3 ± 7.4 nm; PDI = 0.445 ± 0.022; ZP = 43.8 ± 0.3 mV; at 25 °C: Z-Ave = 134.7 ± 0.3 nm; PDI = 0.269 ± 0.017; ZP = 42.7 ± 1.2 mV; at 45 °C: Z-Ave = 231.1 ± 1.4 nm; PDI = 0.464 ± 0.006; and ZP = 44.5 ± 1.6 mV. The purpose of the stability study was to determine the recommended storage temperature for the tested lipid nanoparticles. The most favorable results, i.e., the smallest fluctuations in the values of the determined physicochemical parameters, were obtained for the dispersion stored at room temperature (25 °C). Thus, it was recommended for storing the investigated dispersions. Nevertheless, even at more demanding storage temperatures (4 and 45 °C), the Z-Ave value of 300 nm, indicative of stable dispersion, in this case was not exceeded. The presented results confirmed the stability of the investigated lipid nanoparticles over time, subjected to different temperature conditions, and thus the correct selection of the components of the designed dispersion of solid lipid nanoparticles intended for use as a cosmetic raw material.

### 3.2. Cosmetic Products Enriched with Lipid Nanoparticles—Physicochemical Characterization

#### 3.2.1. pH Test

**Figure 2:**
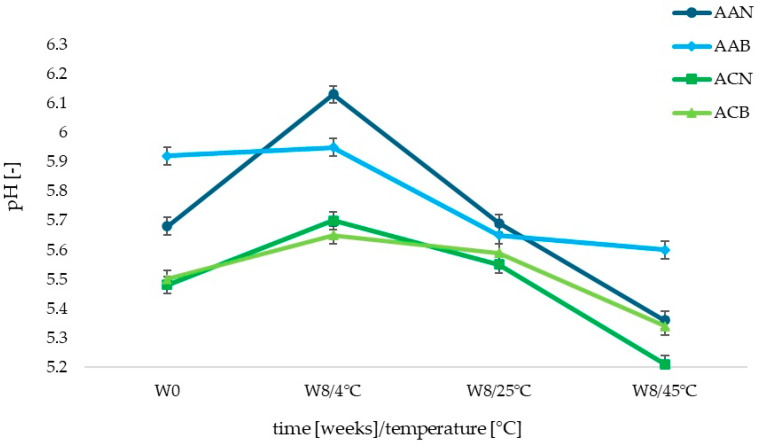
Changes in pH of the cosmetic formulations stored under various temperature conditions (4, 25, 45 °C) for 60 days.

The initial pH values of the cosmetic products tested (W0) were 5.68 ± 0.03 and 5.92 ± 0.02 (for anti-aging formulations, AAN and AAB, respectively); 5.48 ± 0.03 and 5.50 ± 0.01 (for anti-acne formulations, ACN and ACB, respectively) (Figure 2). It can be concluded that the addition of lipid nanoparticle dispersion caused a decrease in the pH values of the tested face creams. This decrease was more noticeable in the case of anti-aging preparations. After 8 weeks of storage (W8), it was observed that for all samples stored at 4 °C, regardless of composition and the presence of SLNs, the pH value increased slightly. For AAN and AAB, the pH was 6.13 ± 0.01 and 5.95 ± 0.02 (an increase of 8% and 1%, respectively), while for ACN and ACB, it was 5.70 ± 0.02 and 5.65 ± 0.04 (an increase of 4% and 3%, respectively). At 25 °C, there were slight fluctuations in the value of the parameter, at an average level of 0.4%. In contrast, at 45 °C, the pH value decreased noticeably. For the AAN and AAB samples, the pH dropped by 0.32 to 5.36 ± 0.03 and 5.60 ± 0.04 (a decrease of 6% and 5%, respectively). As for ACN and ACB formulations, on the other hand, the pH value decreased by 0.27 and 0.21, or 5% and 3%, respectively. 

Based on the analysis of the pH results and the trend of changes in the value of this parameter over time, it was concluded that the presence of lipid nanoparticles does not affect the nature of changes in the pH values of the tested cosmetic formulations. It is also important that active substances such as retinol [31] and peptides are not sensitive to such slight fluctuations in the pH of the cosmetic base, which do not affect the active action of these compounds. It was determined that changes in the pH values of the tested cosmetic formulations were not noticeable enough to affect the processes in the skin and on its surface.

#### 3.2.2. Stability Study by Multiple Light Scattering

The stability of cosmetic products sustained over time and under varying storage temperatures was a basic requirement when testing designed cosmetics enriched with lipid nanoparticles containing retinol and oligopeptide. When evaluating the stability of the tested formulations, the focus was on assessing the level of backscattering (ΔBS) and the Turbiscan Stability Index, TSI, which expresses the total value of all instabilities in the sample [31,32,33]. The use of the multiple light scattering method made it possible to quickly and accurately detect destabilizing phenomena, which are invisible to the naked eye, in the non-transparent and undiluted dispersion system [34].

**Figure 3:**
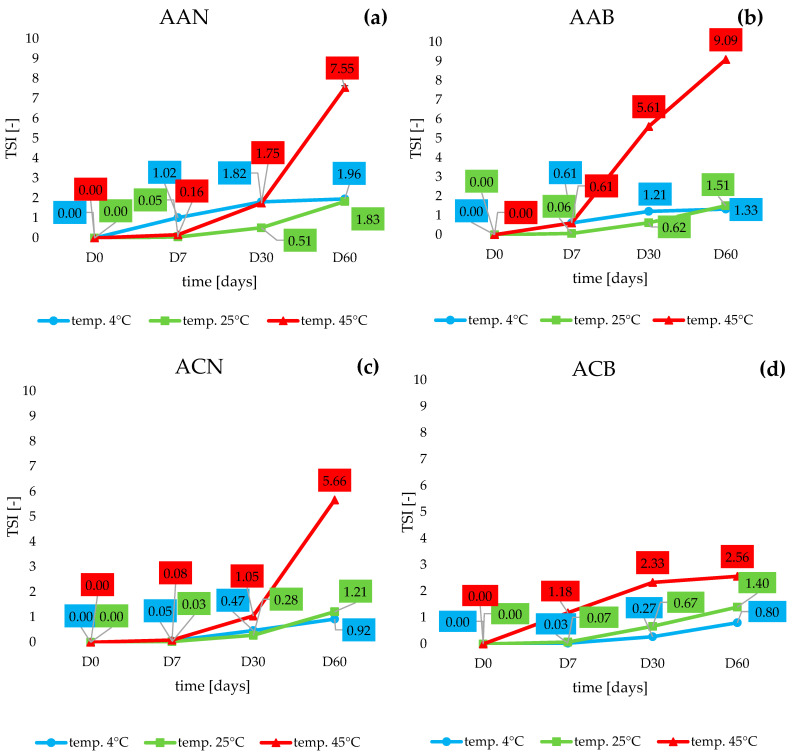
Changes in the Turbiscan Stability Index of the cosmetic formulations ((**a**) AAN; (**b**) AAB; (**c**) ACN; (**d**) ACB) stored under various temperature conditions (4, 25, 45 °C) for 60 days.

The study successfully used the TSI to characterize the instability of the samples. The lower the TSI, the more stable the cosmetic formulation [31,35]. The results of the analysis for two face creams from the AA group (Figure 3a,b) allowed us to conclude that both cosmetic products—both the cream containing lipid nanoparticles (AAN) and the base itself (AAB)—were stable after 60 days at reduced temperature (4 °C) and at 25 °C. The corresponding TSI values did not exceed 2. The TSI value increased only above day 30 for the AAN sample stored at elevated temperature, where an increase in the parameter value was observed from 1.75 (day 30) to 7.55 (day 60). Meanwhile, in the case of the pure cosmetic base (AAB), an increase in TSI value was noted as early as 7 days into the test; the TSI then reached 0.61, before rising to 5.61 after 30 days and to 9.09 after 60 days of the stability test.

In the case of variants of the second tested cosmetic formulation (anti-acne)—ACN (Figure 3c) and ACB (Figure 3d)—the Turbiscan Stability Index did not exceed the value of 1.5 in samples stored for 60 days at 4 °C and 25 °C. A low and acceptable TSI value was also shown by the ACB sample stored at 45 °C; after 60 days, the parameter did not exceed the value of 3. This result testified to the absence of instability-like phenomena [36]. An increase in TSI value was noted only in the ACN formulation between the 30th and 60th days of storage at 45 °C, where an increase from 1.0 to 5.7 was achieved. Nevertheless, it should be remembered that 45 °C is an extreme temperature for cosmetic product samples, and despite the values obtained for the TSI, no instability, such as creaming or sedimentation, visible to the naked eye, appeared in the samples.

**Figure 4:**
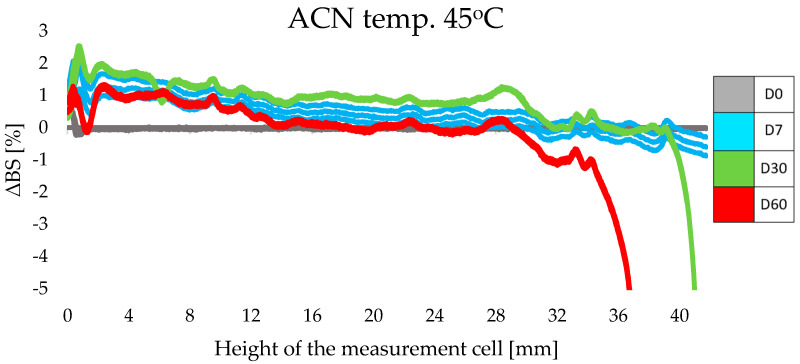
Changes in intensity of the backscattering light for ACN stored at 45 °C for 60 days.

**Figure 5:**
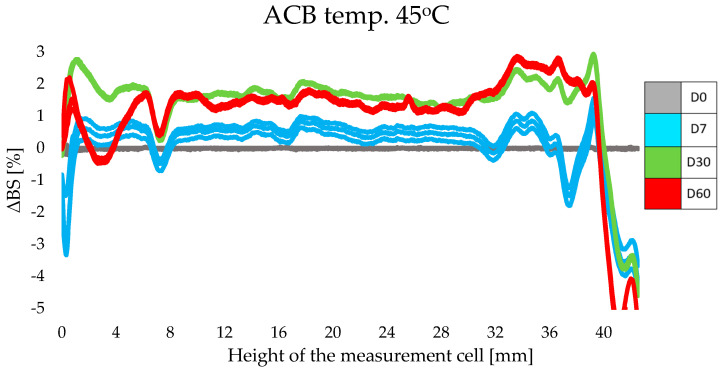
Changes in intensity of the backscattering light for ACB stored at 45 °C for 60 days.

For each sample, the results were also obtained in the form of a stability profile, where the abscissa axis presents the height of the measurement cell, while the ordinate axis shows the intensity of the backscattering light (ΔBS) [14,31,32]. The least favorable ΔBS results were obtained for samples of cosmetic preparations stored at an elevated temperature of 45 °C. The obtained results undoubtedly corresponded to the TSI results described above. The profiles of intensity of the backscattering light indicated the instabilities presented in the upper part of the samples. Examples of ΔBS plots for ACN (Figure 4) and ACB (Figure 5) exposed to 45 °C are shown below. In the case of ACN, the decrease in light intensity at the top of the cell on day 30 may have been the result of changes occurring on the walls of the vessel, where the glass was soiled with the sample during meniscus equilibration on day 0. The thin layer of product that remained on the walls of the cell dried out under the increased temperature and, thus, became apparent on the graphs in the form of a significant decrease in ΔBS intensity. A similar situation was observed for the ACB sample (Figure 5), but the changes in the upper part of the sample were not as intense and noticeable (a decrease in intensity of about 4%).

Qi’s research group claims that particle migration can destabilize the entire system [37]. It is noteworthy, however, that for both samples (ACN and ACB) there was a comparable change in the intensity of backscattered light throughout the sample volume. Significant differences appeared only on the higher parts of the measurement cell. Based on the ΔBS profiles, one can infer the gradually appearing instability of the ACN and ACB formulations. Nevertheless, these values are within the acceptable limit of 10%, which is considered the limit for stable formulations [38].

**Figure 6:**
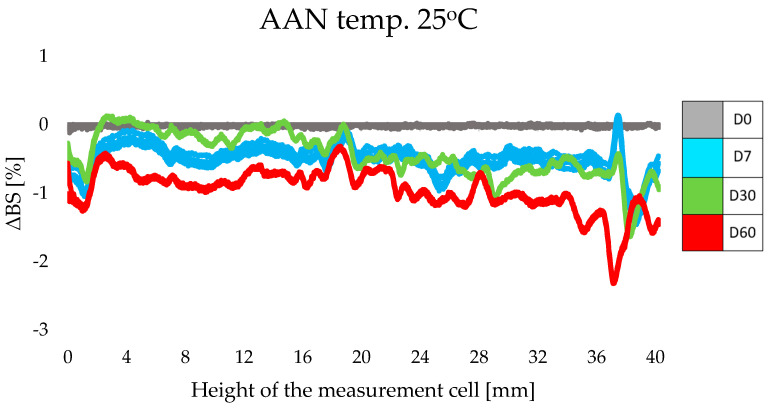
Changes in intensity of the backscattering light for AAN stored at 25 °C for 60 days.

**Figure 7:**
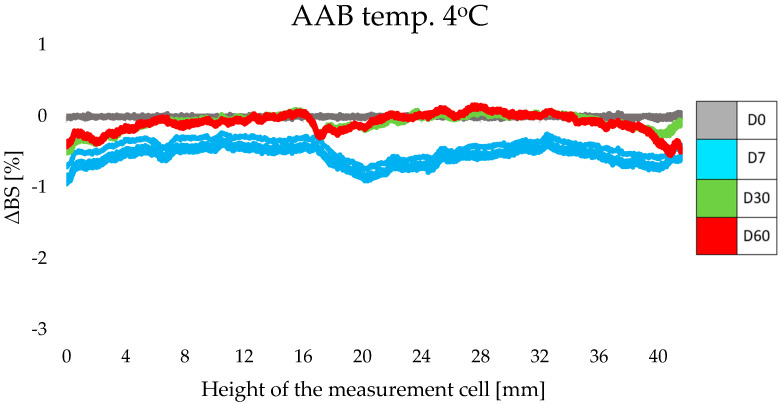
Changes in intensity of the backscattering light for AAB stored at 4 °C for 60 days.

Based on a comparison of the ΔBS plot for the AAN stored at 25 °C (Figure 6) and the ΔBS profile for AAB stored at 4 °C (Figure 7), the assumed storage temperature conditions were considered as recommended, and the mentioned cosmetic formulations were determined to be stable. In both cases, comparable changes in the intensity of backscattered light were observed throughout the sample volume. No changes related to the migration of sample particles toward the top or bottom of the measurement cell were noted. The maximum ΔBS values in the AAN and AAB samples were −2.2% and −1.0%, respectively. These results were compatible with TSI results for analogous samples. The data obtained confirm the stability of the formulations under varying temperature conditions over a period of 60 days. This allowed the tested cosmetic formulations to be approved for further testing.

### 3.3. Cosmetic Products Enriched with Lipid Nanoparticles—Efficacy Testing (In Vivo Tests)

The final stage in the evaluation of the effectiveness of the designed cosmetic formulations and their effect on skin parameters was an in vivo study conducted using non-invasive methods to confirm the claimed properties of the tested cosmetic products. Analysis of the results of the application tests allowed an objective assessment of changes in selected skin parameters, occurring under the influence of the applied cosmetic preparations.

#### 3.3.1. Skin Hydration and Transepidermal Water Loss Measurement

One of the main functions of the epidermis is to maintain proper levels of skin hydration. Effectively ensuring the barrier properties of the epidermis is important because proper hydration affects the healthy appearance of the skin and determines the course of most of the biochemical processes occurring in it [39]. According to Adamski and Kaszuba [23], a too-low water content in the epidermis (less than 10%) causes cracking, roughness, and scaling of the skin. Transepidermal water loss is a natural process that occurs within the structures of the skin. Temperature and humidity can affect the rate at which water evaporates from deeper parts of the skin through the epidermal layer to the external environment [40]. According to Gardien, TEWL is the most relevant physiological parameter of the skin to assess the functionality of the epidermal barrier. It should be mentioned here that repeated and regular application of a cosmetic product containing appropriately selected active substances can have a significant impact on the functioning of the skin barrier [41].

**Figure 8:**
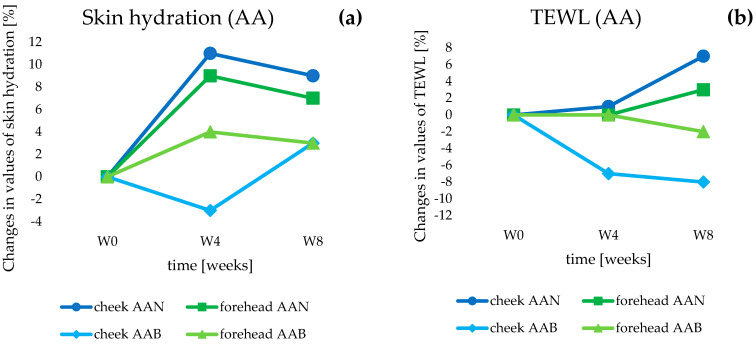
Changes in skin hydration (**a**) and transepidermal water loss (**b**) determined for the anti-aging (AA) cosmetic formulations during in vivo studies.

The AAN cream (anti-aging group), containing 10% SLNs incorporated with retinol and oligopeptide, showed stronger moisturizing properties compared to the anti-aging base (AAB cream), which did not contain nanoparticles with active substances (Figure 8a). After 4 weeks (W4) of using the AAN formulation, skin hydration increased compared to the baseline result (W0), by 11% in the cheek area and by 9% in the forehead area (*p* < 0.05). After another 4 weeks of the study (W8), hydration levels decreased slightly, but the values were higher compared to week 0, at +9% for the cheek and +7%, considering the volunteers’ forehead area (*p* < 0.05). In comparison, in the case of AAB cream, after 4 weeks (W4), there was a 3% decrease in hydration in the cheek area compared to baseline results and a 4% increase in the forehead area (*p* < 0.05). Results obtained at week 8 of the study (W8) indicated only a 3% increase in hydration for both tested application areas (*p* < 0.05). Turning to changes in the value of the TEWL parameter (Figure 8b), the 4-week application (W4) of the AAN cream did not cause statistically significant changes in the value of this parameter. However, significant changes were observed in the 8th week of the study (W8), when TEWL levels increased by 7% (cheek) and 3% (forehead) (*p* < 0.05). Meanwhile, application of the AAB formulation resulted in a reduction in TEWL values of 7% on the cheek and 2% on the forehead at week 4 of the study (W4), and 8% in the cheek area at 8 weeks after the start of application tests (W8) (*p* < 0.05). Changes in TEWL on the forehead skin area were considered statistically insignificant compared to the baseline.

The increase in epidermal hydration and constant TEWL levels in the first stage of the study, i.e., after 4 weeks, indicated the moisturizing properties of the AAN cream. The presence of lipid nanoparticles in the composition of the cosmetic product increased hydration in the lower layers of the epidermis while maintaining the skin’s barrier function. This relationship is due to the ability of lipid nanoparticles to regenerate the outer layers of the skin by building lipid compounds into the intercellular matrix of the stratum corneum [30]. A difference was observed in the level of hydration resulting from the application of AAN and AAB formulation. The changes noted after 8 weeks, namely, a decrease in hydration and a slight increase in transepidermal water loss, were due to the presence of retinol in the AAN cream. Retinol acts as a keratolytic agent: it loosens the bonds between keratinocytes, causing accelerated desquamation of the epidermis and disruption of epidermal continuity [8]. The inclusion of retinol into the lipid matrix of SLNs influenced its prolonged release from the cosmetic base, and thus delayed active action on the skin; hence the conclusion that the decrease in hydration levels observed after a further four weeks of retinol contact with the skin, along with a concomitant increase in TEWL values, may have been the result of the described process. Nevertheless, despite the increase in TEWL values, the skin’s hydration level remained at an optimal level, and its value was even higher than before the start of the study.

**Figure 9:**
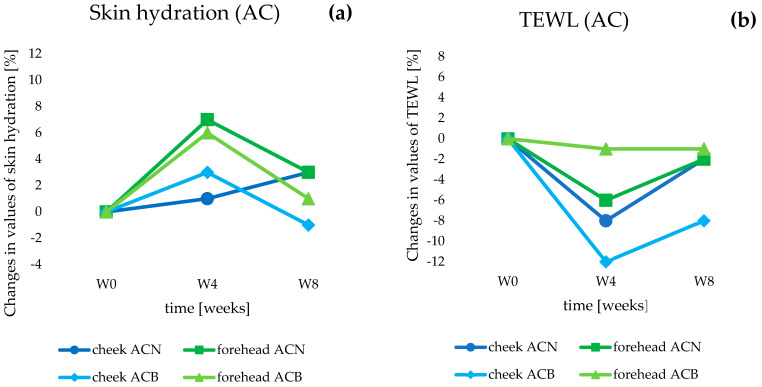
Changes in skin hydration (**a**) and transepidermal water loss (**b**) determined for the anti-acne (AC) cosmetic formulations during in vivo studies.

Using the second group of cosmetic products tested (anti-acne)—the ACN preparation with 5% SLNs containing retinol and oligopeptide—we observed an increase in skin hydration in the first 4 weeks of the study (W4), which amounted to 1% in the cheek area and 7% on the forehead (with respect to baseline values, W0) (*p* < 0.05) (Figure 9a). In contrast, after 8 weeks of application of the cream (W8), the values reached +3% for both tested areas (*p* < 0.05). In comparison, the results recorded for the ACB cream (base without SLNs) were a 3% increase in hydration on the cheek and a 6% increase on the forehead in the 4th week of testing (W4) (*p* < 0.05). After another 4 weeks of ACB application (W8), hydration levels returned almost to baseline values, and the changes were considered statistically insignificant. Considering the TEWL parameters (Figure 9b), for the ACN cream, a decrease in the values of the parameter on both the cheek and forehead was noted, by 8% and 6%, respectively (in week 4, W4) (*p* < 0.05), and a final 2% for both tested areas in the 8th week of application tests (W8) compared to the baseline parameters collected on the day of the start of the study (W0) (*p* < 0.05). In the case of the ACB formulation, we can observe a reduction in TEWL values compared to baseline parameters of 12% in the cheek area (*p* < 0.05), and in the case of the forehead, statistically insignificant changes were noted (W4). Continued use of ACB cream exacerbated the decline in TEWL values, eventually reaching 8% on the cheek at the study’s closing date (W8) (*p* < 0.05), while changes in the forehead area remained insignificant. 

Results obtained after 4 weeks of application of AC cosmetics showed an increase in hydration with a decrease in TEWL, which was due to the presence of SLNs and their previously mentioned occlusive properties. The deterioration in parameters noted at the end of the study was related, as with the AA group products, to the presence of retinol. Importantly, the concentration of retinol in ACN was half that of AAN, which had a significant impact on the keratolytic process and thus on skin hydration levels. Therefore, fluctuations in hydration caused by the desquamation process were much less noticeable, and, importantly, skin parameters did not deteriorate in any case.

#### 3.3.2. Skin Elasticity Measurement

**Figure 10:**
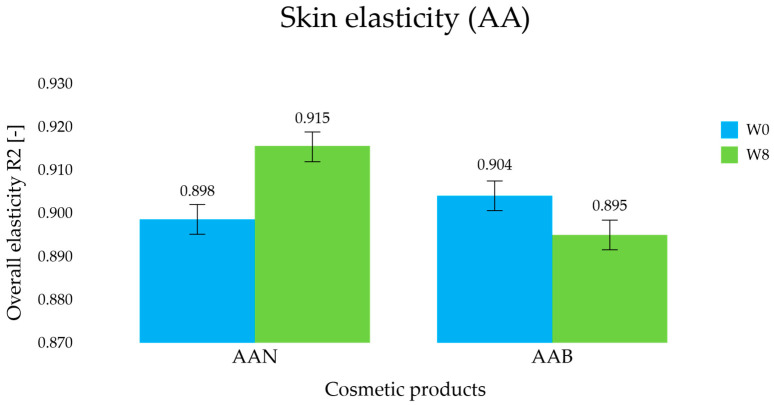
Changes in skin elasticity determined for the anti-aging (AA) cosmetic formulations during in vivo studies.

Determination of biomechanical parameters of the skin, including the overall elasticity (dimensionless coefficient R2), made it possible to assess the “anti-aging” effectiveness of AA cosmetic products. In general, the higher the value of the R2 coefficient and the closer it approaches unity (1), the more elastic and firmer the skin. Turning to the results, the 8-week application of the AAN cream resulted in an increase in R2 from a value of 0.898 ± 0.032 (W0) to 0.915 ± 0.047 (W8) (*p* < 0.05); thus, an improvement in skin elasticity of about 2% was noted (Figure 10). In comparison, for the AAB preparation (without SLNs), the initial results (W0) averaged 0.904 ± 0.024, while the reading on the final day of the study was 0.895 ± 0.038 (W8), making this change in skin elasticity statistically insignificant. 

It was concluded that the use of the AAN cosmetic product for 8 weeks improved the elasticity and firmness of the treated skin. The additive effect of retinol and oligopeptide resulted in improved skin density and elasticity. This could be due to the action of these active substances on skin structures through beneficial effects on collagen and elastin synthesis [8].

#### 3.3.3. Sebum Level Measurement

Cosmetic products from the AC group, due to the presence of active ingredients intended for oily and combination skins, were also tested with the Sebumeter^®^. Due to the nature of the physiological process of sebum secretion by the sebaceous glands, the subjects were required to clean their faces a minimum of one hour before the test and not to apply other emulsion products that could interfere with the reliability of the measurements. According to the principle of measuring the level of sebum secretion, the closer the result obtained is to 0, the greater the skin’s seboregulatory activity [28]. 

**Figure 11:**
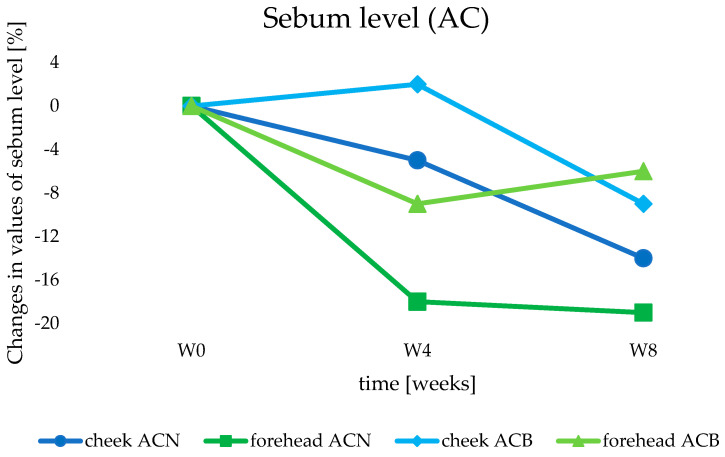
Changes in sebum level determined for the anti-acne (AC) cosmetic formulations during in vivo studies.

During the 8-week use of the ACN and ACB preparation, a significant improvement in the reduction in sebum production was noted in all areas tested (cheek and forehead skin) (Figure 11). The decrease in sebum secretion was noticeable already after 4 weeks of study (W4), especially when the product containing lipid nanoparticles (ACN) was applied to the forehead area of the volunteers. The amount of sebum secreted was then 18% lower than at the start of the study (W0) (*p* < 0.05). This condition continued throughout the rest of the study period, as the final result (W8) was a 19% reduction in sebum in this area (*p* < 0.05). A significant improvement was also observed on the cheek: the use of ACN in the first 4 weeks (W4) reduced the value of the seboregulation-related parameter by 5%, and after 8 weeks (W8), by up to 14% (*p* < 0.05). This parameter was also improved slightly under the application of ACB cream. However, in this case, the reduction reached 9% and 6% in the cheek and forehead areas, respectively (W8) (*p* < 0.05). 

Analysis of the obtained results allowed us to conclude that the formulation of creams from the AC group was adapted to the care of skin with the need to regulate the sebaceous glands and thus the amount of sebum secretion. The choice of texture-forming raw materials additionally supported the action of the active substances retinol and peptide and thus caused an additive effect. After the 8-week study, the decrease in sebum production was more pronounced for the ACN preparation compared to ACB cream; however, the ACB cosmetic also showed a noticeable sebostatic effect. Hence, the conclusion was that even the cosmetic base itself (ACB) devoid of lipid nanoparticles was properly selected for this type of skin with a wide range of problems. The difference in the results obtained for ACN and ACB creams indicated the importance and effectiveness of the active ingredients with which the designed lipid carriers were incorporated. For volunteers with oily and combination skin affected by acne lesions, excess sebum, or local inflammation, AC cosmetic products showed good efficacy. With long-term use, they brought real benefits, reducing both sebum overproduction and skin inflammation.

#### 3.3.4. Skin Macrorelief Parameters

Analysis of the skin macrorelief using profilometric methods made it possible to assess the skin surface in terms of the presence, number, and depth of wrinkles. The study assumed that a decrease in the values of the aforementioned parameters would indicate a reduction in the number and size of wrinkles, thus confirming the anti-wrinkle effect of the tested cosmetic products (from the AA group).

**Figure 12:**
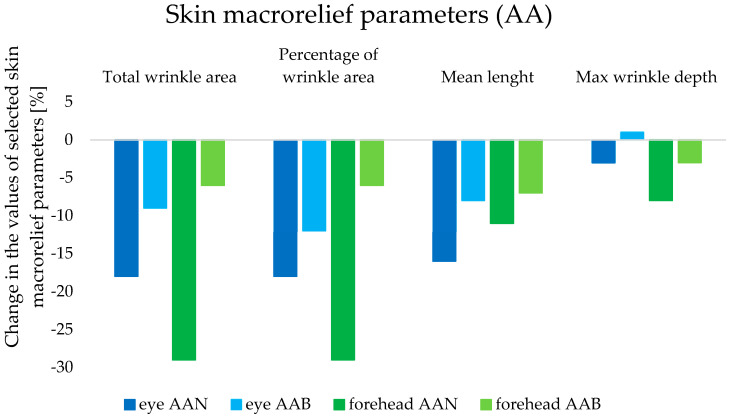
Changes in skin macrorelief parameters determined for the anti-aging (AA) cosmetic formulations (forehead and eye area) during in vivo studies (8 weeks).

The cosmetic product AAN, thanks to the content of retinol and oligopeptide, with which lipid nanoparticles were incorporated, caused a decrease in the number of wrinkles, by 29% in the forehead area and 18% in the eye area (*p* < 0.05), and thus in the percentage of wrinkles in the tested skin area (W8) (Figure 12). In comparison, the results obtained in this regard for the AAB preparation were −6% in the case of the forehead and −9% in the eye area (*p* < 0.05). The difference in the reduction in the average length and maximum depth of wrinkles was also significant. Over the course of 8 weeks (W8), the average length of lines in the tested area, compared to the baseline (W0), was reduced by AAN by 16% on the skin near the eyes and by 11% on the forehead (*p* < 0.05), while the AAB cream caused a reduction in wrinkles of 7% and 8%, respectively (*p* < 0.05). The anti-aging effectiveness of the tested cosmetics was also confirmed by the parameter of maximum wrinkle depth. Under the influence of AAN, its value over 8 weeks was reduced by 8% on the forehead and by 3% in the eye area (*p* < 0.05), while for the AAB product, the changes obtained were not statistically significant. 

Taking into account all parameters of the skin macrorelief, a significant improvement in the condition and overall appearance of the skin was undeniably noticeable, and thus the anti-wrinkle and anti-aging properties of the cosmetic product containing lipid nanoparticles were confirmed.

## 4. Conclusions

Retinol and an oligopeptide (Tyr-D-Ala-Gly-Phe-Leu) were tested to verify their effectiveness as active ingredients in cosmetic formulations. The introduction of these compounds in the form of lipid nanoparticles ensured the acquisition of stable semi-solid formulations characterized by a prolonged effect on the skin over time. It was proven that despite the keratolytic nature of retinol, the tested cosmetic preparations showed properties that maintained an adequate level of epidermal hydration. The presence of lipid nanoparticles positively influenced the barrier function of the skin, thanks to the occlusive properties of SLN, and reduced the undesirable effects of retinol, appearing during the use of retinol in its classic form. The anti-aging effect of a cosmetic product from the AA group was confirmed: the use of AAN cream resulted in a significant improvement in skin elasticity, improving the overall condition of the skin. These properties were attributed to the peptide content in the formulation. The anti-aging effect was also noted during the analysis of the results of the skin macrorelief parameters. The reduction in the number of wrinkles and the reduction in their length and depth over 8 weeks of application of the AAN formulation undoubtedly indicated the effective action of the cosmetic product. The synergistic effect of oligopeptide and retinol was proven for the anti-acne (AC) product; a significant seboregulatory function of the active substances contained in this formulation and their anti-acne effect were observed. The conducted research confirmed the effectiveness of the tested cosmetic products while adhering to the assumptions of the current trend of “skin minimalism”. Measurable effects (in vivo study) of the developed semi-solid preparation enriched with solid lipid nanoparticles were achieved while using the lowest effective concentrations of active ingredients (retinol and oligopeptide) in the cosmetic formulation.

## Data Availability

The data presented in this study are available by authors upon request. The general procedure of the emulsions’ formation is only available to non-professionals.

## References

[1] Charoenputtakhun P., Opanasopit P., Rojanarata T., Ngawhirunpat T. (2014). All-trans retinoic acid-loaded lipid nanoparticles as a transdermal drug delivery carrier. Pharm. Dev. Technol..

[2] Yadav N., Khatak S., Vir U., Sara S. (2013). Solid lipid nanoparticles—A review. Int. J. Appl. Pharm..

[3] Resende D.I., Ferreira M.S., Sousa-Lobo J.M., Sousa E., Almeida I.F. (2021). Usage of synthetic peptides in cosmetics for sensitive skin. Pharmaceuticals.

[4] Janiszewska J. (2014). Naturalne peptydy przeciwdrobnoustrojowe w zastosowaniach biomedycznych. Polimery.

[5] Olejnik A., Nowak I., Schroeder G. (2009). Peptydy jako nowe syntetyczne składniki preparatów kosmetycznych. Receptory molekularne—Właściwości i Zastosowanie.

[6] Lipotec® (2020). ‘Leuphasyl®’.

[7] Errante F., Ledwoń P., Latajka R., Rovero P., Papini A.M. (2020). Cosmeceutical Peptides in the Framework of Sustainable Wellness Economy. Front. Chem..

[8] Pawłowska M., Marzec M., Nowak I., Jankowiak W. (2023). Retinoids. Chemical diversity for health and beauty. Przem. Chem..

[9] Bojarowicz H., Płowiec A. (2010). Wpływ witaminy A na kondycję skóry. Probl. Hig. Epidemiol..

[10] Marona H., Gunia A., Pękala E. (2010). Retinoidy—Rola w farmakoterapii w aspekcie komórkowego mechanizmu działania. Farm. Pol..

[11] Tratnjek L., Jeruc J., Romih R., Zupančič D. (2021). Vitamin A and retinoids in bladder cancer chemoprevention and treatment: A narrative review of current evidence, challenges and future prospects. Int. J. Mol. Sci..

[12] Baumann L. (2009). Cosmetic Dermatology.

[13] Wissing S.A., Müller R.H. (2003). Cosmetic applications for solid lipid nanoparticles (SLN). Int. J. Pharm..

[14] Lasoń E., Ogonowski J. (2011). Stałe Nanocząsteczki Lipidowe—Charakterystyka, zastosowanie i otrzymywanie. Chemik.

[15] Hallan S.S., Sguizzato M., Esposito E., Cortesi R. (2021). Challenges in the physical characterization of lipid nanoparticles. Pharmaceutics.

[16] Ferreira R., Napoli J., Enver T., Bernardino L., Ferreira L. (2020). Advances and challenges in retinoid delivery systems in regenerative and therapeutic medicine. Nat. Commun..

[17] Anil L., Kannan K. (2018). Microemulsion as drug delivery system for peptides and proteins. J. Pharm. Sci. Res..

[18] Almeida A.J., Runge S., Müller R.H. (1997). Peptide-loaded solid lipid nanoparticles (SLN): Influence of production parameters. Int. J. Pharm..

[19] Kołaczek A. (2015). Przegląd metodpielęgnacji skóry dojrzałej. Kosmetol. Estet..

[20] Marwicka J., Gałuszka A. (2021). Use of vitamin preparations in the skin care process. Aesthetic Cosmetol. Med..

[21] Bernat M., Matysek-Nawrocka M., Cioczek W. (2016). Składniki aktywne w kosmetykach przeciwstarzeniowych. Aesthetic Cosmetol. Med..

[22] Noszczyk M. (2012). Kosmetologia Pielęgnacyjna i Lekarska.

[23] Adamski Z., Kaszuba A. (2008). Dermatologia dla Kosmetologów.

[24] ZetaSizer Nano Series, Zetasizer Nano Series User Manual; Zeta Potential Theory; Malvern Instruments Ltd.: Worcestershire, UK, 2004.

[25] Courage+Khazaka (2017). Tewameter TM 300.

[26] Courage+Khazaka (2020). Corneometer CM 825.

[27] Courage+Khazaka (2019). Cutometer MPA 580.

[28] Courage+Khazaka (2016). Sebumeter® SM 815.

[29] Courage+Khazaka (2016). Visioline VL 650-Quantiride.

[30] Dąbrowska M., Nowak I. (2021). Lipid nanoparticles loaded with selected iridoid glycosides as effective components of hydrogel formulations. Materials.

[31] Lee S.C., Yuk H.G., Lee D.H., Lee K.E., Hwang Y.I., Ludescher R.D. (2002). Stabilization of retinol through incorporation into liposomes. J. Biochem. Mol. Biol..

[32] Dąbrowska M.A. (2019). Optymalizacja Właściwości Fizykochemicznych Oraz Aplikacyjnych Formulacji Kosmetycznych Zawierających Wybrane Glikozydy Irydoidowe.

[33] (2013). Lab Expert, Turbiscan ®—Manual instruction.

[34] Gagliardi A., Paolino D., Costa N., Fresta M., Cosco D. (2021). Zein- vs PLGA-based nanoparticles containing rutin: A comparative investigation. Mater. Sci. Eng. C.

[35] Luo M., Qi X., Ren T., Huang Y., Keller A.A., Wang H., Wu B., Jin H., Li F. (2017). Heteroaggregation of CeO_2_ and TiO_2_ engineered nanoparticles in the aqueous phase: Application of turbiscan stability index and fluorescence excitation-emission matrix (EEM) spectra. Colloids Surfaces A Physicochem. Eng. Asp..

[36] Gagliardi A., Paolino D., Iannone M., Palma E. (2018). Sodium deoxycholate-decorated zein nanoparticles for a stable colloidal drug delivery system. Int. J. Nanomed..

[37] Qi X., Dong Y., Wang H., Wang C., Li F. (2017). Application of Turbiscan in the homoaggregation and heteroaggregation of copper nanoparticles. Colloids Surfaces A Physicochem. Eng. Asp..

[38] Celia C., Trapasso E., Cosco D., Paolino D., Fresta M. (2009). Turbiscan Lab^®^ Expert analysis of the stability of ethosomes^®^ and ultradeformable liposomes containing a bilayer fluidizing agent. Colloids Surf. B Biointerfaces.

[39] Kilpatric-Liverman L. (2014). Mechanisms of Skin Hydration. Cosmetic Science and Technology.

[40] Machado M., Salgado T.M., Hadgraft J., Lane M.E. (2010). The relationship between transepidermal water loss and skin permeability. Int. J. Pharm..

[41] Gardien K.L.M., Baas D.C., de Vet H.C.W., Middelkoop E. (2016). Transepidermal water loss measured with the Tewameter TM300 in burn scars. Burns.

